# FXS-Like Phenotype in Two Unrelated Patients Carrying a Methylated Premutation of the *FMR1* Gene

**DOI:** 10.3389/fgene.2018.00442

**Published:** 2018-11-02

**Authors:** Esperanza Fernández, Elena Gennaro, Filomena Pirozzi, Chiara Baldo, Francesca Forzano, Licia Turolla, Francesca Faravelli, Denise Gastaldo, Domenico Coviello, Marina Grasso, Claudia Bagni

**Affiliations:** ^1^Center for Human Genetics, KU Leuven, Leuven, Belgium; ^2^VIB & KU Leuven Center for Brain & Disease Research, Leuven, Belgium; ^3^Laboratorio di Genetica Umana, Ospedali Galliera, Genoa, Italy; ^4^Clinical Genetics Department, Borough Wing Guy’s Hospital, Guy’s and St Thomas’ NHS Foundation Trust, London, United Kingdom; ^5^S.S.D. Genetica Medica, Ospedali Galliera, Genoa, Italy; ^6^U.O.S. Genetica Medica, Azienda ULSS 2, Treviso, Italy; ^7^Clinical Genetics Department, Great Ormond Street Hospital, London, United Kingdom; ^8^Department of Fundamental Neurosciences, University of Lausanne, Lausanne, Switzerland; ^9^Department of Biomedicine and Prevention, University of Rome Tor Vergata, Rome, Italy

**Keywords:** FMRP, *FMR1* mRNA, CGG expansion, fragile X syndrome, mosaicism

## Abstract

Fragile X syndrome (FXS) is mostly caused by two distinct events that occur in the *FMR1* gene (Xq27.3): an expansion above 200 repeats of a CGG triplet located in the 5′UTR of the gene, and methylation of the cytosines located in the CpG islands upstream of the CGG repeats. Here, we describe two unrelated families with one FXS child and another sibling presenting mild intellectual disability and behavioral features evocative of FXS. Genetic characterization of the undiagnosed sibling revealed mosaicism in both the CGG expansion size and the methylation levels in the different tissues analyzed. This report shows that in the same family, two siblings carrying different CGG repeats, one in the full-mutation range and the other in the premutation range, present methylation mosaicism and consequent decreased FMRP production leading to FXS and FXS-like features, respectively. Decreased FMRP levels, more than the number of repeats seem to correlate with the severity of FXS clinical phenotypes.

## Introduction

Fragile X syndrome (FXS) (MIM# 300624) is the most common form of inherited intellectual disability and the most common monogenic form of autism spectrum disorders (ASDs). The typical features of FXS include anxiety, hyperactivity, attention deficit disorder, speech perseveration, stereotypical movements and impulsive behavior (Martin and Bell, 1943; Dykens et al., 1989; Hodapp et al., 1990; Sullivan et al., 2006; Cordeiro et al., 2011). FXS is caused by a dynamic expansion of the polymorphic CGG triplet in the 5′UTR of the fragile X mental retardation (*FMR1*) (MIM# 309550) gene, located on the X chromosome (Fu et al., 1991; Verkerk et al., 1991). Alleles containing >200 CGG triplets generally lead to DNA methylation and abnormal heterochromatinization due to altered methylation and histone deacetylation (full mutation) (Oberle et al., 1991; Coffee et al., 1999). This epigenetic mechanism results in the silencing of *FMR1* gene and the consequent loss of its product, the fragile X mental retardation protein (FMRP). FMRP is an RNA-binding protein ubiquitously expressed that functions as a translational repressor in the brain and has a key role in the regulation of local protein synthesis at synapses (Bagni et al., 2012; Richter et al., 2015).

Based on the number of CGG triplets and the methylation status, several *FMR1* alleles are present in the human population: the unmethylated alleles, containing 5–54 repeats; the premutation alleles containing 55–200 repeats that are typically not methylated; the full-mutation alleles containing >200 repeats that are typically methylated; and the more rare unmethylated full-mutation alleles (Pirozzi et al., 2011; Bagni et al., 2012). The unmethylated full-mutation alleles are transcriptionally active with an FMRP production that negatively correlates with the repeat number. Such a decrease is due to a deficit in translation efficiency (Kenneson et al., 2001; Primerano et al., 2002).

In individuals carrying premutation alleles the *FMR1* mRNA levels are increased and progressively accumulates in inclusions (Tassone et al., 2004; Arocena et al., 2005) that result in the fragile X-associated Tremor Ataxia syndrome (FXTAS) (MIM# 300623), a late onset autonomic disorder with cognitive dysfunction (Jacquemont et al., 2004). FXTAS patients have cognitive decline with some individuals carrying premutation alleles with large expansion of the triplets who might have a mild intellectual disability as a result of increased *FMR1* mRNA and slightly reduced FMRP amount compared to normal alleles. FXTAS affects at least 33% of premutation males, with an age-dependent increased incidence, and 5–10% of premutation females (Hagerman and Hagerman, 2004; Greco et al., 2008), while 12–28% of female premutation carriers develop fragile X-associated premature ovarian insufficiency (FXPOI) (Allingham-Hawkins et al., 1999; Sherman, 2000).

Since the discovery of the gene (Verkerk et al., 1991), few cases have been reported carrying unmethylated expansion > 200 CGG with no intellectual disability or a mild FXS phenotype and apparently normal levels of FMRP (Tassone et al., 2001; Pretto et al., 2013; Pretto D.I. et al., 2014). This phenomenon is explained by somatic mosaicism (Genc et al., 2000; Pretto D.I. et al., 2014), highlighting the FXS genetic heterogeneity. In this context, three scenarios are possible: full-mutation alleles coexist with premutation alleles in different cell types or in different cells of the same cytotype (size mosaicism); cells where methylation patterns are different on all the alleles (methylation mosaic); or cells with a combination of the two previous possibilities (size and methylation mosaicism). The pattern of mosaicisms does not exhibit any familiar association but may show a high frequency, reaching up to 41% of the FXS patients (Nolin et al., 1994). Mosaicism can impact the penetrance of the disorder, in fact the CGG size plus the methylation status of full-mutation mosaics seem to negatively correlate with cognitive functions (McConkie-Rosell et al., 1993; Hagerman et al., 1994; Schmucker et al., 1996; Wohrle et al., 1998; Helderman-van den Enden et al., 1999). It has been reported that FXS males show greater development of adaptive skills in mosaic cases than in full-mutation cases, suggesting that phenotypic severity can be influenced by the presence of mosaicism (Cohen et al., 1996).

Premutation alleles found in somatic cells have been considered very stable in contrast to germline cells where they show more instability with significant variations in repeat size (Nolin et al., 1999; Tassone et al., 1999a). However, somatic repeat expansions have recently been reported to occur in the premutation mouse model, in a human premutation lymphoblastoid cell line as well as in brain regions and blood of a 91 CGG repeat premutation human carrier (Lokanga et al., 2013). Furthermore, although premutation alleles are generally unmethylated, a small percentage of cells might carry a premutation allele with a percentage of methylation that affects FMRP levels in the different cell types (Allingham-Hawkins et al., 1996; Tassone et al., 1999b). Here we report two unrelated Caucasian families, each with one individual with FXS and one sibling presenting an FXS-like phenotype.

## Results

### Clinical Evaluation

Case 1 is a 10-year-old male who showed normal speech and motor development in the first year of life. During development, he showed signs of hyperactivity, attention deficit, stereotypies and “learning deficits” mainly in logical areas. At 8.5 years of age, he underwent a thorough neuropsychological evaluation through a Wechsler Intelligence Scale for Children (WISC-III) test. WISC-III revealed a disharmonic profile with lower scores in the language area (VIQ = 88; PIQ = 117; TIQ = 102). Certain abilities such as understanding, verbal fluency and auditory attention were categorized as not appropriate for his age (Supplementary Table S1). No other health problems were identified.

Case 2 is a 21-year-old male who showed normal motor development in the first year of life. He exhibited a significant delay in speech development with first words at 18 months, and almost exclusive sign language until 4 years of age. Attention deficit and hyperactivity were noted at a very early age. As a toddler, he showed mild *genu valgum*, and he developed scoliosis during middle childhood. By the time he started elementary school, he exhibited learning difficulties, prompting a referral for a neuropsychological evaluation where specific support was requested. At the age of 10 he exhibited inadequate abilities compared to children of his age (Supplementary Table S1). He showed particular difficulties in the spatio-temporal abilities, reproduction of geometrical figures and segmental control. Memorization and mental calculation were also inadequate for age. Proofs in writing, reading and speech showed dysorthography, inadequate metalinguistics, reading speed and comprehension, difficulties in the pronunciation of some phonemes and atypical swallowing. At 14 years of age, attention and concentration deficits were persistent and he showed a low self-esteem. Additionally, impairments in reading, writing, and memorization were evident. At 16 years of age the patient presented with main difficulties in attention and short-term memory and was diagnosed with dysorthography and dyscalculia. A WISC-R test showed an IQ at the lower limits of the normal range.

### Molecular Analysis

**FIGURE 1:**
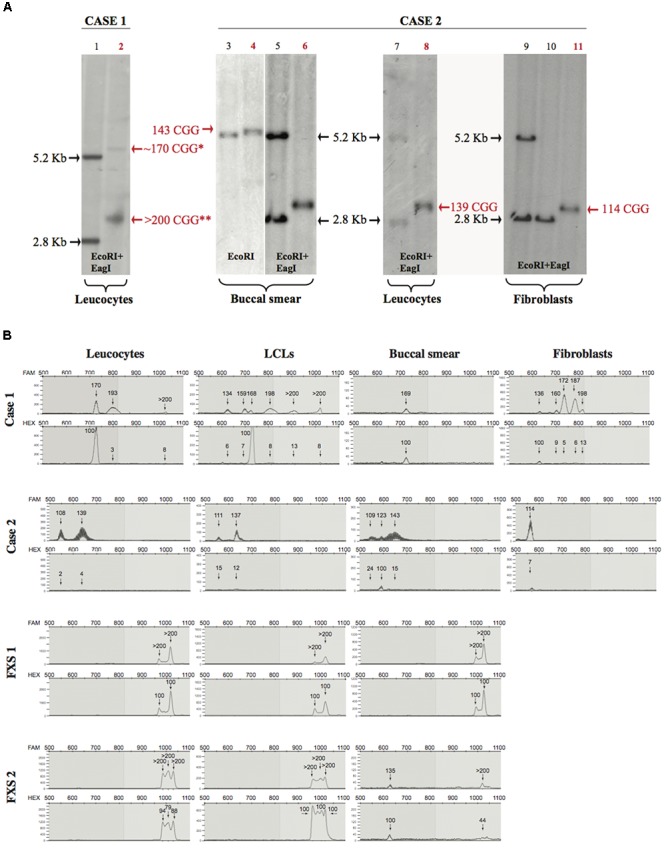
Southern blot and mPCR analysis. **(A)** Southern blots of Case 1 and Case 2 (lane numbers for these samples are shown in red). Left panel (control in lane 1 and Case 1 in lane 2), analysis upon EcoRI-EagI DNA digestion from leucocytes. Right panels (controls in lanes 3, 5, 7, 9, 10 and Case 2 in lanes 4, 6, 8, 11) analysis upon EcoRI or EcoRI-EagI DNA digestion. Relative number of triplets is noted next to the bands; red text refers to number of triplets belonging to Case 1 and Case 2. ^∗^ is used to indicate a methylated premutation (lane 2), while two stars indicate the >200 CGG unmethylated allele. It is notable that, in Case 1 (lane 2), the 170 premutation allele is methylated while the expansion over 200 CGG (full mutation) is unmethylated; in Case 2, the methylated premutation allele of 123 repeats it is not detectable by Southern blot (lane 6 buccal smear). **(B)** Electropherograms representing CGG repetition alleles (top panel) and methylation percentages (bottom panel) relative to different tissues for Case 1, Case 2, FXS 1, and FXS 2 samples.

On the basis of the neuropsychological evaluation of the two cases (Case 1 and Case 2) and the presence of two siblings with FXS (respectively, designated as FXS 1 and FXS 2), we performed a molecular analysis of the *FMR1* gene. For Case 1, a Southern blot performed on DNA extracted from peripheral blood showed the presence of an unmethylated full-mutation (delta > 600 bp) with a mosaic of premutation and methylated alleles (Figure 1A). For Case 2, a Southern blot performed on DNA extracted from buccal smear, peripheral blood (leucocytes), and skin (fibroblasts) revealed the presence of a premutation (delta 200–300 bp) in the three cytotypes analyzed (Figure 1A).

The CGG expansion analysis was further evaluated in leucocytes, lymphoblastoid cell lines (LCLs), fibroblasts, and buccal smear samples from both individuals using methylation PCR (mPCR, Table 1). Both cases showed size and methylation mosaicism within the premutation range. Case 1 had a CGG triplet region that spanned from 134 to >200 repeats with a percent of methylation that varied from 3 to 100% in the different alleles (Table 1 and Figure 1B). Leucocytes and LCLs contained the rare unmethylated full-mutation allele, one unmethylated premutation allele on the high repetition range (193 repeats in leucocytes and 198 repeats in LCLs) and one fully methylated premutation allele of 170 repeats (5.4 Kb). The latter allele was also conserved in the buccal smear. LCLs also contained two other unmethylated premutation alleles. Fibroblasts exhibited the widest variety of CGG length and methylation status when compared to the other tissues. Fibroblasts contained a fully methylated premutation allele of 136 repeats and four unmethylated premutation alleles, with only one of them conserved in leucocytes and LCLs (Table 1; Figure 1B). Thus, molecular analysis of Case 1 revealed a clear inter- and intra-mosaicism, with alleles conserved among different tissues that coexisted with others that were tissue-specific. The sibling of this proband, FXS 1, presented with a clear FXS phenotype and exhibited a full-mutation allele in all the tissues tested (Table 1; Figure 1B).

**Table 1:** CGG expansion and methylation status in different tissues.

			Leucocytes		Buccal smear		Fibroblasts		Lymphoblastoid cell lines	
	Sample	Phenotype	CGG repeats	Methylation (%)	CGG repeats	Methylation (%)	CGG repeats	Methylation (%)	CGG repeats	Methylation (%)
**Family 1**	CTRL 4	Non-affected	32	7	n.a.	n.a.	n.a.	n.a.	32	17
	Case 1	FXS-like	170	100	169	100	136	100	134	6
			193	3			160	9	159	7
			>200	8			172	5	168	100
							187	6	198	8
							198	13	>200	8–13
	FXS 1	FXS	>200	100	>200	100	n.a.	n.a.	>200	100

**Family 2**	Case 2	FXS-like	108	2	109	24	114	7	111	15
			139	4	123	100			137	12
					143	15				
	FXS 2	FXS	>200	80–100	135	100	n.a.	n.a.	>200	100
					>200	44				

**Unrelated controls**	CTRL 1	Control	30	2	n.a.		n.a.		n.a.	
	CTRL 2	Control	29	14	29	6	n.a.		n.a.	
	CTRL 3	Control	n.a.		n.a.		n.a.		30	6
	CTRL 5	Control	n.a.		n.a.		n.a.		32	3
	CTRL 6	Control	n.a.		n.a.		n.a.		29	9
	CTRL 7	Control	n.a.		n.a.		n.a.	0	n.a.	n.a.
	CTRL 8	Control	n.a.		n.a.		n.a.	0	n.a.	n.a.

**Unrelated FXS**	FXS 3^∗^	FXS	n.a.		n.a.		500	100	n.a.	n.a.


*The table reports 2 families with FXS individuals (FXS 1 and FXS 2), FXS-like individuals (Case 1 and Case 2), non-affected control of family 1 (CTRL4), and 7 unrelated unaffected individuals (CTRL 1, 2, 3, 5, 6, 7, 8). The *FMR1* alleles were detected by DNA analysis from whole blood, lymphoblastoid cell lines, buccal smears, and fibroblasts. For each tissue, the CGG triplet expansion and the methylation status (%) are listed. n.a.: non-available data. ^∗^FXS unrelated individual (84E0275), see Jacquemont et al. (2018).*

Case 2 exhibited another case of inter- and intra-mosaicism but with more conserved alleles among the tissues compared to Case 1. Analysis of Case 2 uncovered one unmethylated premutation allele that spanned from 108 to 143 CGG repeats in leucocytes, LCLs, buccal smear and fibroblasts (Table 1 and Figure 1B). Case 2 presented with another unmethylated premutation allele that expanded 139, 137, and 143 repeats (∼3 Kb) in leucocytes, LCLs, and buccal smear, respectively. The buccal smear also displayed a fully methylated premutation allele, with this being the only tissue tested that showed a methylation mosaicism (Table 1 and Figure 1B). In contrast to Case 1, this proband did not show any full-mutation alleles in the cells tested, showing an intra- and inter-mosaicism due to *FMR1* premutation alleles. This is a rare case of an individual carrying *FMR1* methylated premutation alleles with FXS-like phenotypes which has rarely been seen before (Tassone et al., 2000; Farzin et al., 2006; Chonchaiya et al., 2009, 2012). The sibling of this proband, FXS 2, was diagnosed with a clear FXS phenotype and exhibited a full-mutation allele in leucocytes and LCLs but also showed mosaicism in the buccal smear with one partially methylated full-mutation allele coexisting with a fully methylated premutation allele (Table 1 and Figure 1B).

In both cases, the percentage of methylation and CGG triplet size varied among all the cell types tested, as observed by Southern blot and mPCR assay (Table 1 and Figure 1). Importantly, the alleles clearly showed an absence of linearity between the methylation status and the triplet expansion that has not been reported before in the classification of FXS alleles (Bagni et al., 2012). The FXS-like phenotype of both probands could be explained by the presence of a methylated premutation allele in different cell types.

**FIGURE 2:**
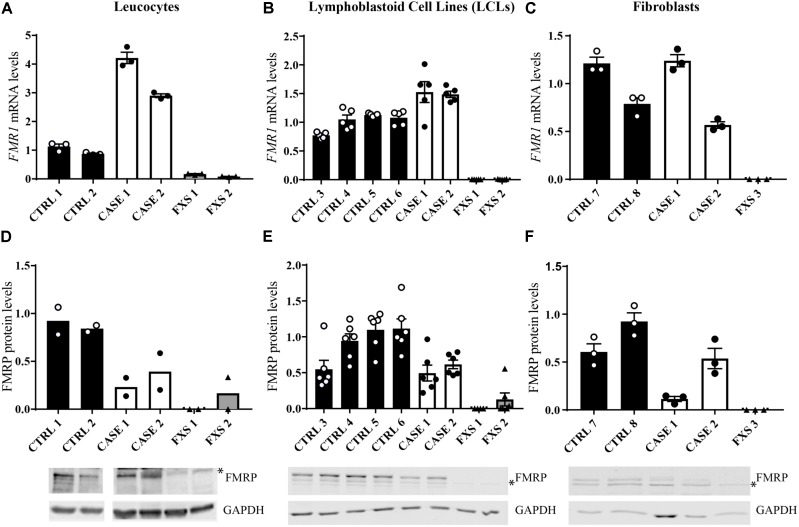
Analysis of *FMR1* mRNA and FMRP expression in different tissues. **(A–C)** Quantitative RT-qPCR detecting *FMR1* mRNA relative expression in the tissues tested, normalized with the housekeeping *HPRT1* mRNA [*n* = 3–5, technical replicates, independent experiments for **(B,C)**]. **(D–F)** Quantification of FMRP levels compared to GAPDH levels [*n* = 2–6 technical replicates, independent experiments for **(E,F)**]. The error bars indicate the standard error. Lower panels, representative Western blots from the different cell types analyzed. ^∗^ indicates a non-specific signal which has been subtracted in the quantification.

In order to investigate whether the CGG size and methylation mosaicism affect FMRP production, we tested the expression levels of *FMR1* mRNA and FMRP protein in different cell types and tissues (leucocytes, LCLs, and fibroblasts) and compared them with the FXS siblings and unaffected controls. As an average of the tissues tested, the probands (Case 1 and Case 2) showed higher levels of *FMR1* mRNA (Figures 2A–C) with decreased protein production compared to controls (Figures 2D–F). Levels of mRNA and protein expression are directly and indirectly related to the allele size and the percentage of *FMR1* gene methylation. In leucocytes, for Case 1, *FMR1* mRNA expression levels were found to be around 4-fold higher than controls (Figure 2A) with a reduction of around 60% in protein levels (Figure 2D); while Case 2 had a 3-fold increase in mRNA levels and a corresponding 50% reduction of the protein levels (Figures 2A–D), consistent with the presence of premutation alleles. In LCLs and fibroblasts, for both cases *FMR1* mRNA levels were higher or within the normal range, respectively (Figures 2B,C), although FMRP levels were still at least 50% reduced compared to controls (Figures 2E,F), consistent with previous findings in premutation allele carriers. Of note, while the presence of several alleles reflects the genetic heterogeneity of cell type or tissue, mRNA and protein levels of cultured cells might reflect a selective growth of cells carrying specific alleles (Khajavi et al., 2001).

## Materials and Methods

### Subjects

Patients and their relatives were recruited through the Galliera Hospital in Genova, Italy and their biological samples were stored in the “Galliera Genetic Bank.” Sex and age-matched controls were obtained from the “Galliera Genetic Bank” (Galliera Hospital, Genoa, Italy); and from the “Cell line and DNA Biobank from patients affected by Genetic Diseases” (IRCCS Giannini Gaslini, Genoa, Italy) (Filocamo et al., 2014; Baldo et al., 2016). Participants provided written informed consent for clinical and molecular analyses and for the publication of the results on scientific journals; the protocols of the study were approved by the relevant ethics committee. In this study we included five male participants with FXS mutations belonging to the full mutation (*n* = 3; namely FXS 1, FXS 2, and FXS 3) and methylation and size mosaicism (*n* = 2; namely Case 1 and Case 2), and eight control subjects (CTRL 1–8). It was not possible to obtain skin biopsies from the individuals FXS 1 and FXS 2, therefore an unrelated FXS case (FXS 3), was analyzed for western blot and RT-qPCR. FXS 3 fibroblasts (84E0275) were kindly provided by Dr. Robert Willemsen and described in (Jacquemont et al., 2018). Individual ages ranged from 6 to 47 years (median ± SD = 20.5 ± 0.5 years). Control samples (CTRL 7 and 8) were purchased by Coriell Institute^1^.

### Establishment of Lymphoblastoid Cell Lines

Ten milliliters of blood were drawn from a peripheral vein, and lymphoblasts were isolated on a Ficoll-diatrizoate density gradient (Ficoll-Paque; Pharmacia). B-lymphoblasts were immortalized by incubation with supernatant containing Epstein–Barr virus. After immortalization, B-lymphoblasts were grown for 10–14 days in RPMI 1640 medium (Gibco), supplemented with 2 mM L-glutamine, 100 U/mL penicillin, 100 mg/mL streptomycin (Gibco), and 10% FBS.

### Establishment of Primary Fibroblast Cell Lines

Written consent was obtained both from patients and control subjects before acquiring skin biopsies according to the procedure of the “Galliera Genetic Bank” (Ethics Committee Reference No. 8/2015). Fibroblasts were isolated from 3 mm biopsies using standard procedures. Cells were manually dissociated and grown in RPMI1640 supplemented with 10% fetal calf serum (Thermo Fisher Scientific) in 100-mm dishes. Cell lines were cultured at 37°C with 5% CO2 and media was replaced every 3–4 days. Fibroblast cultures were passaged three–five times prior to the collection of DNA, RNA, protein isolation, or cryopreservation.

### Southern Blot Analysis

DNA from whole blood was isolated using QIAsymphony robot (QIAGEN, Heilden, Germany). DNA from LCLs, fibroblast cell lines and buccal smears was isolated using the QIAamp DNA Blood mini Kit (QIAGEN, Heilden, Germany) and extracted using phenol–chloroform. In order to assess the CGG repeat length, DNA was analyzed by Southern blot as previously described (Gatta et al., 2013).

### PCR Assay for the Detection of Methylation Status in the *FMR1* Gene (mPCR)

DNA samples were analyzed for methylation status and CGG repeat length using the AmplideX *FMR1* mPCR reagents (Asuragen, Austin, TX, United States) as described previously (Grasso et al., 2014). Briefly, 8 μL of 10–30 ng/μL DNA samples were premixed with two plasmids: a digestion control and PCR reference control. This premixture was separately aliquoted to a control or methylation-sensitive digestion reaction. Restriction digestion, PCR, and capillary electrophoresis were performed as previously described by (Chen et al., 2011). All alleles were detected using FAM-labeled primers, but only the proportion of the protected methylated allele was available for PCR using HEX-labeled primers. Lack of methylation at HpaII sites resulted in digestion and thus no amplification.

### *FMR1* mRNA and FMRP Protein Expression Levels

Total RNA from leucocytes, lymphoblastoid and fibroblasts cell lines was isolated using Trizol (Qiagen, Valencia, CA, United States). Total RNA was reverse transcribed into cDNA using the MMLV reverse transcriptase (Life Technologies). *FMR1* cDNA was amplified by real time PCR (RT-qPCR; Applied Biosystems). Technical replicates were performed for each cell line (*n* = 3 for leucocytes, *n* = 5 for LCLs, *n* = 3 for fibroblasts). *FMR1* expression levels were determined relative to the reference gene *HPRT1*, using the 2ˆ(-ddct) method and comparing each sample to the average value of the controls. The following primers were used: *HPRT1* (Forward TGCTGAGGATTTGGAAAGGGT; Reverse TCGAGCAAGACGTTCAGTCC), *FMR1* (Forward TGTCAGATTAGATTCCCACCTCCTG; Reverse TAACCACCAACAGCAAGGCT).

Protein expression levels were detected by Western blotting. Antibodies against FMRP (rAMII) were previously described (Ferrari et al., 2007), and antibodies against GAPDH were purchased from two different sources (Developmental Studies Hybridoma Bank and Thermo Fisher Scientific). Quantification of the FMRP levels was obtained calculating the FMRP/GAPDH ratio and comparing each sample to the average value of the controls. Standard error of the mean (SEM) is shown.

## Discussion

These two FXS-like cases show variability of alleles with different methylation patterns, thus illustrating somatic instability. In Case 1, the range of alleles crossed the premutation range (200 CGG repeats) in two of the tissues tested, with most of the alleles predominantly clustered in the premutation range. This individual exhibited allele methylation, but in contrast to previous mosaicism cases (Pretto D.I. et al., 2014), the percentage of allele methylation did not increase with the CGG repeat number, but varied between alleles of similar size (Table 1). This suggests that CGG repeat expansion and stability are not the only causative elements for the methylation status of the *FMR1* gene. Instead, other epigenetic mechanisms that are cell type-specific are likely to be involved, as previously suggested (Burman et al., 1999; Wohrle et al., 2001). Case 1 and Case 2, in fact are two examples of complex genetic patterns with an effect on *FMR1* mRNA and FMRP production that underlie the penetrance of complex FXS phenotypes. Because epigenetic mechanisms might affect FMRP expression independently from the CGG expansion, this factor should also be considered while estimating the patient’s prognosis, see also (Stoger et al., 2011). Of note, the size of the CGG repeats was recently shown to significantly associate with the degree of clinical features (Pretto D. et al., 2014).

Somatic mosaicism of repeat length is present in other repeat expansion disorders such as Huntington’s disease, spinocerebellar ataxia and myotonic dystrophy (MD) (Liquori et al., 2001; Richards, 2001). In these disorders, the mosaicism is prominent and tends to be age-dependent, expansion-biased, and highly tissue-specific (Monckton and Caskey, 1995; Monckton et al., 1999; Sato et al., 1999; Kennedy and Shelbourne, 2000). It is likely that somatic mosaicism contributes, at least in part, to the late age of onset in most of the disorders associated with unstable DNA expansions. In MD, the degree of expansion increases throughout life, representing a risk for clinical progression (Martorell et al., 1995; Martorell et al., 1998; Fortune et al., 2000). It would be of high interest to follow whether the two FXS-like mosaicism cases studied here show the epigenetic variation during later adulthood and whether there is a correlation with the progression of clinical features. Indeed, the different degree of CGG size and methylation status of the identified alleles suggests that other cell types from complex organs such as the brain may also exhibit size and methylation instability, and may account for the phenotypes of the patients. Three patients carrying the fragile X premutation and full mutation have been found to show somatic instability in different brain regions (D’Gama et al., 2015). Furthermore, as Case 1 and 2 carry premutation and/or full-mutation unmethylated alleles, they might eventually develop FXTAS. The onset of this disorder and the worsening of the symptoms with age could be due to a prevalence of certain alleles over others.

Genetic analysis of tissues and cells of premutation carriers as well as mosaic patients at later stages of adulthood could help understand the pathophysiology of this disorder as well as correlate the genetic modifications with the clinical phenotypes. We cannot exclude the possibility that the pattern of premutation alleles could result from a contraction of the full-mutation unstable allele and that both alleles coexist in the same tissue. Although this is a genetic event rarely described, few cases of contraction of a maternal high premutation/full mutation *FMR1* allele during transmission have been reported (Yrigollen et al., 2014; Miranda et al., 2015; Maia et al., 2017). More unique it is the case of contraction of an expanded *FMR1* unstable allele to a normal size, recently reported (Manor et al., 2017).

The molecular mechanisms that give rise to somatic mosaicism are not yet fully understood. Somatic mosaicism seems to accumulate through multiple small mutations (Monckton et al., 1995) that require the mismatch repair machinery (Manley et al., 1999; van den Broek et al., 2002; Savouret et al., 2003; Wheeler et al., 2003) and is independent of cell division (Fortune et al., 2000; Kennedy and Shelbourne, 2000; Gomes-Pereira et al., 2001). It has been suggested that variation in the expansion rate observed in MD patients (Martorell et al., 1998) as well as variation in disease severity (Hunter et al., 1992) might naturally occur due to natural environmental modifiers. In this regard, therapies that target the somatic repeat expansion may have a general utility to treat these disorders. Notoriously, chronic exposure to certain agents induced significant changes in the expansion rate of the CAG–CTG repeat sequence (Gomes-Pereira and Monckton, 2004) by increasing or reducing the number of repeats. FXS mosaic cases result from a combination of alleles with variable CGG repeats as well as an independent methylation status. Exposure to modifiers that limit the somatic repeat expansion in combination with demethylating agents is a very attractive chemical gene therapy, particularly since small molecule drugs can be tested in several cells and tissues derived from the patient.

## Concluding Remarks

We report two atypical cases of genetic inter- and intra-somatic mosaicism that carry several patterns of *FMR1* alleles. In both cases, the alleles show a variable methylation status and different CGG triplet expansion within the premutation range and with a lack of conservation linearity within the tissues tested. The unmethylated full-mutation allele also co-existed with premutation alleles in two of the tissues of Case 1. The diversity of premutation alleles had a direct impact in mRNA and protein levels: both cases expressed higher levels of mRNA and lower levels of protein than controls. The existence of these mosaic cases reveals that genetic variations in *FMR1* gene, that affect FMRP expression levels, may underlie cognitive impairment in similar neurodevelopmental disorders. These cases highlight the importance of performing FXS clinical tests in blood and in other tissues. Furthermore, the test should be expanded to patients presenting cognitive impairment without a clear diagnosis.

## Ethics Statement

This study was approved on August 7, 2012 by President of the Ethical Committee: Prof. Walter Van den Bogaert, Reference No. S54626/ML8532; samples provided by Telethon Network of Genetic Biobanks (Project No. GTB12001) have been distributed in agreement with principles stated in informed consent form approved by Liguria Ethical Committee N.8/2015 on September 14, 2015.

## Author Contributions

EF and FP performed the RT-qPCR and Western blotting and wrote the manuscript. EG, ChB, FFo, LT, FFa, and DC recruited the families, collected the samples, and performed the characterization of the CGG expansion and DNA methylation status. DG contributed to the revision of the paper with RT-qPCR and Western blotting for Figure 2. ClB and MG directed the work and wrote the manuscript. All the authors approved the manuscript before submission.

## Conflict of Interest Statement

The authors declare that the research was conducted in the absence of any commercial or financial relationships that could be construed as a potential conflict of interest.

## Footnotes

1 https://www.coriell.org
