# Supplementary material for: FXS-Like Phenotype in Two Unrelated Patients Carrying a Methylated Premutation of the FMR1 Gene
Source: Front Genet. 2018 Nov 2;9:442. doi: 10.3389/fgene.2018.00442 (PMC6224343; doi:10.3389/fgene.2018.00442)
Supplement: Supplementary file 1 [file Table_1.docx]

| **Clinical picture** | **Case 1** | **Case 2** |
| --- | --- | --- |
| Pregnancy | Blood loss at gestational week 9 | Pre-eclampsia |
| Gestational weeks | 38 | 38 |
| Developmental features: |  |  |
| Motor | Normal | Normal |
| Speech | Normal | Delayed |
| Behaviour | ADHD, stereotypies | ADHD |
| IQ | Normal (TIQ = 102) | Lower limits of normal range |
| Neuropsychological evaluation | ADHD | ADHD |
|  | Relatively low WISC score in the language area (VIQ = 88) | Difficulties in the spatio-temporal abilities |
|  | Understanding, verbal fluency, auditory attention inadequate for chronological age | Impairment in reading and writing |
|  |  | Impairments in memorization |
|  |  | Dysorthography |
|  |  | Dyscalculia |
|  |  | Anxiety |
| Orthopedic problems |  | Scoliosis and mild genu valgum |
| Peculiar facial features | Large, prominent ears (97^th^ c) | None |

**Supplementary Table 1.** Clinical features of FXS-like Case 1 and Case 2.
